# Non-invasive Scores and Serum Biomarkers for Fatty Liver in the Era of Metabolic Dysfunction-associated Steatotic Liver Disease (MASLD): A Comprehensive Review From NAFLD to MAFLD and MASLD

**DOI:** 10.1007/s13679-024-00574-z

**Published:** 2024-05-29

**Authors:** Farah Abdelhameed, Chris Kite, Lukasz Lagojda, Alexander Dallaway, Kamaljit Kaur Chatha, Surinderjeet S. Chaggar, Maria Dalamaga, Eva Kassi, Ioannis Kyrou, Harpal S. Randeva

**Affiliations:** 1 Warwickshire Institute for the Study of Diabetes, Endocrinology and Metabolism (WISDEM), University Hospitals Coventry and Warwickshire NHS Trust, Coventry, CV2 2DX UK; 2 Warwick Medical School, University of Warwick, Coventry, CV4 7AL UK; 3 School of Health and Society, Faculty of Education, Health and Wellbeing, University of Wolverhampton, Wolverhampton, WV1 1LY UK; 4 Centre for Sport, Exercise and Life Sciences, Research Institute for Health & Wellbeing, Coventry University, Coventry, CV1 5FB UK; 5 Chester Medical School, University of Chester, Shrewsbury, SY3 8HQ UK; 6 Clinical Evidence-Based Information Service (CEBIS), University Hospitals Coventry and Warwickshire NHS Trust, Coventry, CV2 2DX UK; 7 Department of Biochemistry and Immunology, University Hospitals Coventry and Warwickshire NHS Trust, Coventry, CV2 2DX UK; 8 Institute for Cardiometabolic Medicine, University Hospitals Coventry and Warwickshire NHS Trust, Coventry, CV2 2DX UK; 9 Sowe Valley Primary Care Network, Forum Health Centre, Coventry, CV2 5EP UK; 10 Department of Biological Chemistry, Medical School, National and Kapodistrian University of Athens, Athens, Greece; 11 First Department of Propaupedic and Internal Medicine, Endocrine Unit, Laiko Hospital, National and Kapodistrian University of Athens, 11527 Athens, Greece; 12 Aston Medical School, College of Health and Life Sciences, Aston University, Birmingham, B4 7ET UK; 13 College of Health, Psychology and Social Care, University of Derby, Derby, DE22 1GB UK; 14 Laboratory of Dietetics and Quality of Life, Department of Food Science and Human Nutrition, School of Food and Nutritional Sciences, Agricultural University of Athens, 11855 Athens, Greece

**Keywords:** Fatty liver, Metabolic dysfunction-associated fatty liver disease, MAFLD, Metabolic dysfunction-associated steatotic liver disease, MASLD, Non-alcoholic fatty liver disease, NAFLD, Biomarkers, Obesity

## Abstract

**Purpose of Review:**

The prevalence of non-alcoholic fatty liver disease (NAFLD) is rapidly increasing worldwide, making it the leading cause of liver related morbidity and mortality. Currently, liver biopsy is the gold standard for assessing individuals with steatohepatitis and fibrosis. However, its invasiveness, sampling variability, and impracticality for large-scale screening has driven the search for non-invasive methods for early diagnosis and staging. In this review, we comprehensively summarise the evidence on the diagnostic performance and limitations of existing non-invasive serum biomarkers and scores in the diagnosis and evaluation of steatosis, steatohepatitis, and fibrosis.

**Recent Findings:**

Several non-invasive serum biomarkers and scores have been developed over the last decade, although none has successfully been able to replace liver biopsy. The introduction of new NAFLD terminology, namely metabolic dysfunction-associated fatty liver disease (MAFLD) and more recently metabolic dysfunction-associated steatotic liver disease (MASLD), has initiated a debate on the interchangeability of these terminologies. Indeed, there is a need for more research on the variability of the performance of non-invasive serum biomarkers and scores across the diagnostic entities of NAFLD, MAFLD and MASLD.

**Summary:**

There remains a significant need for finding valid and reliable non-invasive methods for early diagnosis and assessment of steatohepatitis and fibrosis to facilitate prompt risk stratification and management to prevent disease progression and complications. Further exploration of the landscape of MASLD under the newly defined disease subtypes is warranted, with the need for more robust evidence to support the use of commonly used serum scores against the new MASLD criteria and validation of previously developed scores.

## Introduction

**Fig.1:**
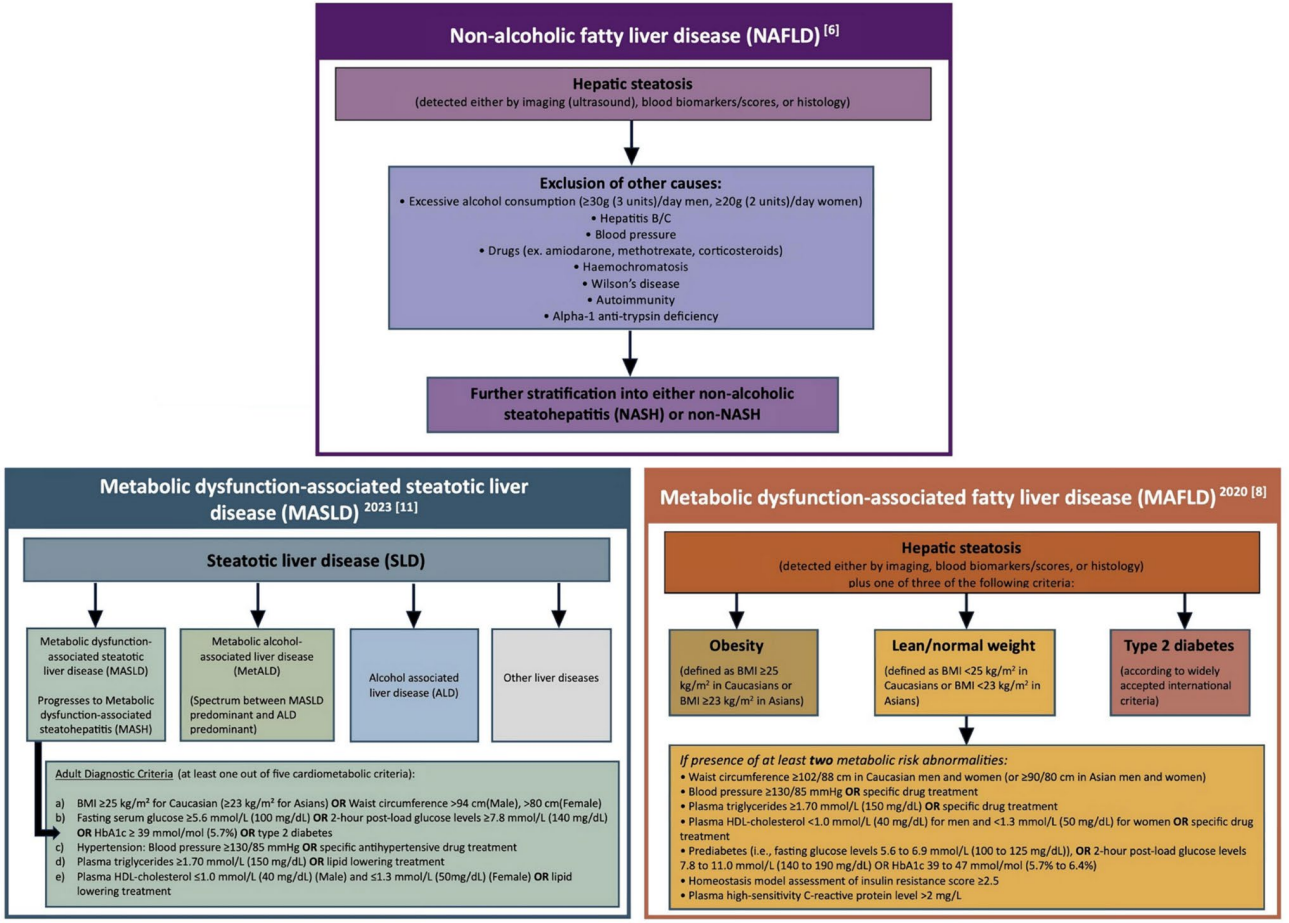
Summary of the diagnostic criteria for NAFLD, MAFLD and MASLD [6, 8••, 11••]

Non-alcoholic fatty liver disease (NAFLD) is the most common cause of chronic liver disease worldwide and the leading cause of liver-related morbidity and mortality [1]. The global prevalence estimates for NAFLD in the general adult population has increased from around 25% in the early 2000s to 32% over the last decade, affecting up to two billion individuals worldwide, mirroring the obesity and diabetes epidemic [2, 3]. Furthermore, it is now recognised that NAFLD has a bidirectional relationship with metabolic syndrome, namely obesity, type 2 diabetes mellitus (T2DM), dyslipidemia, and hypertension, and often occurs in tandem with one or more of these components [4, 5]. Remarkably, the prevalence of NAFLD in adults with one or more of these cardio-metabolic diseases rises steeply to over 60–75% [6, 7]. Given the association of NAFLD with cardio-metabolic diseases and risk factors, an expert panel in 2020 proposed to update the nomenclature for NAFLD to metabolic dysfunction-associated fatty liver disease (MAFLD) [8••]. This was proposed to underscore the heterogeneous entity of NAFLD and its association with metabolic risk factors that may co-exist with other liver diseases, such as alcohol-related liver disease [8••]. Although the MAFLD terminology has gained some global acceptance, concerns were raised regarding the mixing of various aetiologies, as well as regarding the preservation of the term “fatty” in the updated nomenclature, since this term was considered stigmatising [9, 10•]. To address these concerns, a multi-society Delphi statement was published in 2023, proposing to further revise the relevant nomenclature to metabolic dysfunction-associated steatotic liver disease (MASLD) [11••]. In addition to omitting the term “fatty” from the new nomenclature, MASLD is defined by hepatic steatosis and the presence of at least one cardio-metabolic risk factor [11••]. The diagnostic criteria for the three nomenclatures are summarised in Fig. 1.

Following the recent proposal of the MASLD nomenclature/definition, increasing but still limited data explore MASLD in comparison to NAFLD and/or MAFLD, with concerns raised regarding whether the evidence accumulated under the NAFLD nomenclature/definition can be directly extrapolated to MASLD [10•, 12, 13••]. For example, in a recent study involving 6429 patients diagnosed with NAFLD, 99% met the criteria for MASLD [12]. This study concluded that while MASLD may carry a slightly increased mortality risk, it exhibits comparable clinical profiles and thresholds for non-invasive tests across both conditions, suggesting the potential for the interchangeable use of these terminologies [12]. However, a cross-sectional study involving 500 participants who had undergone liver ultrasound and vibration-controlled transient elastography as part of a routine check, showed that the MASLD criteria captures more lean patients compared to the MAFLD definition [14•]. Despite the profound similarities in their respective diagnostic criteria (Fig. 1), in that study, individuals with MAFLD and MASLD displayed a more unfavorable metabolic profile than those with only MASLD. Consequently, following the introduction of the term MASLD, researchers have advocated for more adaptable editorial practices instead of strict adherence to the proposed MASLD nomenclature. It is essential to emphasize that each of these three classifications—NAFLD, MAFLD, and MASLD—carries distinct definitions. Hence, ensuring accurate differentiation between those is crucial for the pertinent scientific literature [10•, 15•].

Given the challenges arising from the different nomenclatures and definitions in the assessment of fatty liver disease, limited reviews have addressed the complexity of the evolving landscape of this disease with respect to serum biomarker scores for all three nomenclatures (NAFLD, MAFLD, and MASLD). The aim of this review is to explore the challenges posed by the new disease nomenclatures to evaluate the diagnostic performance of non-invasive scores and biomarkers across all three diagnostic entities, analysing the existing knowledge to assess their role in the assessment of steatosis, steatohepatitis, and fibrosis. We will also discuss novel advances in this field in terms of multiomic-based biomarkers. The present review will apply the terms NAFLD, MAFLD and MASLD interchangeably as NAFLD/MAFLD/MASLD regarding pathophysiology aspects and the general clinical evaluation of the disease. However, when referencing specific clinical studies on non-invasive scores or serum biomarkers, we will adhere to the terminology or definition used in the corresponding study (i.e., NAFLD, MAFLD, or MASLD).

## Key Pathophysiologic Aspects of NAFLD/MAFLD/MASLD

NAFLD/MAFLD/MASLD is characterised by excessive deposition of fat within hepatocytes (> 5% of hepatocytes with macrovesicular steatosis containing visible intracellular triglycerides or steatosis affecting at least 5% of the liver volume/weight). Although NAFLD diagnosis is considered one of exclusion of alternative liver disease aetiologies (e.g., hepatitis due to viruses, alcohol, and drugs), the diagnosis of MAFLD and MASLD recognizes that in many patients both metabolic and alcohol-related components contribute to hepatic injury and that this condition is based on the presence of metabolic dysfunction rather than on the absence of other liver conditions (Fig. 1) [12, 13••, 14•]. As with NAFLD and non-alcoholic steatohepatitis (NASH), the MAFLD/MASLD disease spectrum represents a continuum from simple hepatic steatosis to metabolic-associated steatohepatitis (MASH), which may progress to fibrosis, leading to cirrhosis and hepatocellular carcinoma in advanced cases. Understanding the dynamic progression of NAFLD/MAFLD/MASLD is paramount for optimizing patient management. This has prompted the development of numerous non-invasive scores and serum biomarkers for the diagnosis of the different underlying pathologies (e.g., for steatosis, steatohepatitis and fibrosis). Contrary to prior assumptions, the progression of NAFLD/MAFLD/MASLD may follow a nonlinear pattern, which challenges the convention that only a small percentage of patients with steatosis would advance to NASH/MASH or develop cirrhosis and end-stage liver disease [13••]. Current knowledge indicates a more complex reality, where the disease may progress, regress, or remain stable [13••, 14•]. As such, identifying patients with advancing steatohepatitis or fibrosis becomes crucial, given the profound impact of fibrosis on liver-related outcomes, such as progression to cirrhosis and overall mortality.

Overall, NAFLD/MAFLD/MASLD imposes a significant clinical, social, and economic burden on both patients and healthcare systems given its potentially severe hepatic and extra-hepatic complications. Patients with NAFLD/MAFLD/MASLD, particularly those with NASH/MASH, are at a higher risk of developing cardiovascular disease, cancer, and infectious diseases [13••]. Notably, the presence of even simple steatosis increases the risk of developing T2DM and hypertension twofold [16, 17]. As such and given their shared risk factors, cardiovascular disease is the most common cause of mortality in these patients, accounting for 40% of the cases [18]. Moreover, decompensated cirrhosis and its associated complications (e.g., encephalopathy and variceal bleeding) occurs at an annual rate of 4% in patients with cirrhosis [19]. Hepatocellular carcinoma constitutes another serious complication of NAFLD/MAFLD/MASLD progression with poor prognostic outcomes [13••]. The complex interplay between NASH/MASH and fibrosis, particularly in advanced stages, makes it challenging to delineate the individual contributions of each to adverse outcomes [20]. Nonetheless, the direct association of steatohepatitis and hepatic fibrosis with adverse outcomes (e.g., liver and cardiovascular events, as well as metabolic complications, and all-cause mortality) is clear and well established in the literature, emphasizing the critical need for early recognition and management of steatohepatitis and hepatic fibrosis in NAFLD/MAFLD/MASLD [21, 22•, 23•, 24]. The pathophysiological mechanisms behind these associations have not been fully elucidated, although these appear to be partially related to the pronounced local and systemic pro-inflammatory profile on a background of worsening insulin resistance [23•, 25]. This underscores the urgency of accurate and timely diagnosis for addressing liver-related complications and also mitigating the compounded risk of cardiovascular disease associated with NAFLD/MAFLD/MASLD and metabolic syndrome.

In routine clinical practice, the key concern in patients with NAFLD/MAFLD/MASLD is the differentiation between NASH/MASH and simple steatosis, and the ascertainment of advanced fibrosis. Currently, liver biopsy remains the "imperfect” gold standard for the diagnosis of fibrosis. Given its intra- and inter-observer limitations in addition to the risks of complications associated with the procedure, and the large number of at-risk patients, liver biopsy is neither a cost-effective nor a practical modality for screening and risk stratification of NAFLD/MAFLD/MASLD patients. This has driven the search for non-invasive methods for early NAFLD/MAFLD/MASLD diagnosis, staging of liver fibrosis, and monitoring disease progression. In this context, the aim of this review is to explore the performance, benefits, and limitations associated with non-invasive serum biomarkers and scores in the diagnosis of steatosis, quantification of steatohepatitis, and detection of advanced liver fibrosis.

## Clinical Evaluation of NAFLD/MAFLD/MASLD

**Fig.2:**
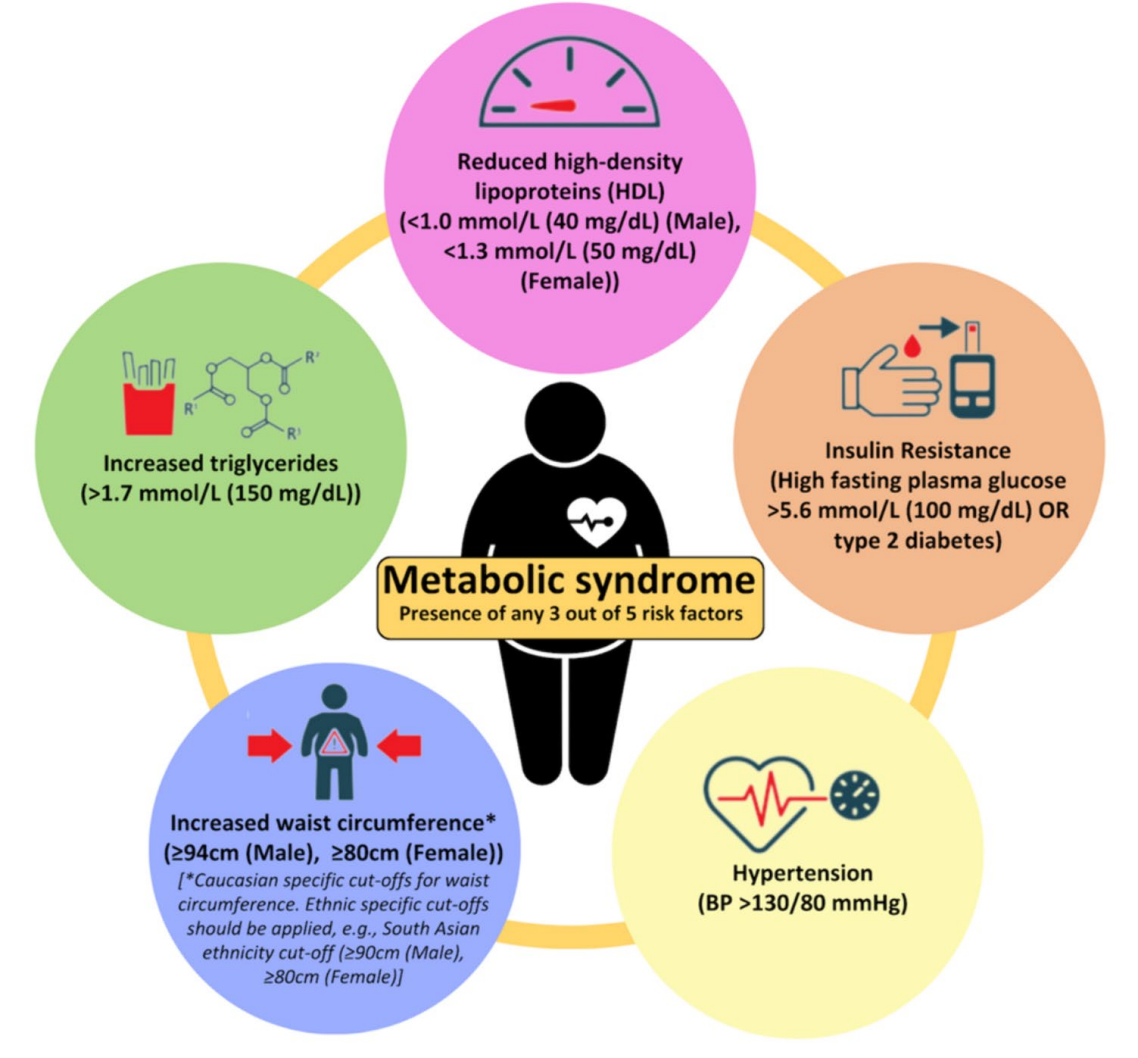
Current diagnostic criteria for metabolic syndrome according to the 2009 joint statement of the International Diabetes Federation Task Force on Epidemiology and Prevention, the National Heart, Lung, and Blood Institute the American Heart Association, the World Heart Federation, the International Atherosclerosis Society, and the International Association for the Study of Obesity [24, 25]

Following the introduction of the aforementioned new nomenclatures, the diagnostic approach of NAFLD/MAFLD/MASLD in routine clinical practice should consider the diagnostic criteria presented in Fig. 1. Moreover, since specific pharmacological treatments for NAFLD/MAFLD/MASLD are now being developed/approved, whilst metabolic dysfunction/comorbidity is present in most cases, the management of NAFLD/MAFLD/MASLD relies crucially on the treatment of the present metabolic syndrome components in order to prevent or reverse progression to NASH/MASH [11••]. The current criteria for the diagnosis of the metabolic syndrome are presented in Fig. 2 [24, 25]. Notably, NAFLD/MAFLD/MASLD can also occur in patients without obesity (Fig. 1), hence the possibility of NASH/MASH should not be overlooked in lean patients presenting with lower cut-offs for the routinely utilised obesity-related indices, such as waist circumference and body mass index (e.g., BMI < 25 kg/m^2^) [26]. Moreover, approximately 80% of patients with steatosis exhibit no specific symptoms or biochemical abnormalities, hence NAFLD/MAFLD/MASLD represents a mostly silent disease until the development of complications, and thus may pose a diagnostic challenge at least initially [27]. In this context, and since liver biopsy for diagnosing NAFLD/MAFLD/MASLD has the aforementioned limitations, a combination of non-invasive methods, including serum biomarkers, scores, and imaging, are applied in clinical practice, depending on availability, in order to diagnose/stage steatosis, steatohepatitis and hepatic fibrosis. A detailed review of non-invasive imaging for NAFLD/MAFLD/MASLD, such as ultrasound-based elastography [e.g., transient elastography (Fibroscan), and shear wave elastography] and magnetic resonance imaging [e.g., magnetic resonance elastography (MRE), and MRI proton density fat-fraction (MRI-PDFF)], has been presented elsewhere, and is beyond the aims of this review [28–31].

## Steatosis Related Scores

Several scores have been reported in the literature to detect and grade steatosis, including the Fatty Liver Index (FLI), Hepatic Steatosis Index (HSI), SteatoTest, and NAFLD Liver Fat Score (LFS) which are described in the following sections. Table 1 summarises the diagnostic performance of these scores from selected key studies for hepatic steatosis assessment [32, 33•, 34]. However, it should be noted that the incorporation of these scores into routine clinical practice is generally restricted due to limitations regarding their diagnostic efficacy (e.g., variability in patient cohorts and validation against liver biopsy and imaging methods) [33•]. As such, there is a debate regarding the added diagnostic information gained from these compared to routinely conducted laboratory and imaging studies in patients suspected with NAFLD/MAFLD/MASLD. Furthermore, there has also been evidence demonstrating underperformance of these scores in patients with co-morbidities such as T2DM, further limiting their routine applicability [35].

**Table 1:** Selected key studies on the diagnostic performance of serum biomarker scores for hepatic steatosis assessment

**Non-invasive steatosis scores**	**Population [Ref.]**	**Cut-offs**	**Sensitivity**	**Specificity**	**AUROC**
**Fatty Liver Index** (BMI, waist circumference, triglycerides, GGT)	n = 496 with and without SLD [36]	< 30 ≥ 60	87% 61%	64% 86%	0.84
	n = 324 with NAFLD [38]	> 60	76%	87%	0.83
	n = 278 with MAFLD [39]	> 30	71%	71%	0.78
	n = 447 with MAFLD [40]	> 30	71%	71%	0.79
	n = 374 with MASLD [22•]	< 30 ≥ 60	99% 96%	10% 23%	0.70
**Hepatic Steatosis Index** (BMI, diabetes, AST/ALT ratio)	n = 5362 with NAFLD [41]	< 30 > 36	93% 46%	40% 92%	0.81
	n = 119 children with severe obesity [42]	-	67%	62%	0.68
	n = 324 with NAFLD [38]	> 42	61%	93%	0.81
	n = 447 with MAFLD [40]	> 33	72%	71%	0.78
	n = 374 with MASLD [22•]	< 30 > 36	100% 96%	5% 44%	0.80
**SteatoTest** (ALT, α2-macroglobulin, apolipoprotein A-I, haptoglobin, total bilirubin, GGT, cholesterol, triglycerides, glucose, age, gender, and BMI)	n = 884 with CLD (CHC, CHB, ALD, NAFLD) [43]	> 0.3	90%	54%	0.80
	n = 494 with severe obesity [44]	> 0.38	90%	45%	0.80
	n = 600 with NAFLD [45]	> 0.48	-	-	0.82
**NAFLD Liver Fat Score** (metabolic syndrome, type 2 diabetes, FSI, AST, and AST/ALT ratio)	n = 470 with NAFLD [46]	> -0.64	86%	71%	0.86
	n = 324 with NAFLD [38]	> 0.16	65%	87%	0.80
	n = 374 with MASLD [22•]	< -0.64 ≥ 1.26	92% 70%	46% 96%	0.87

*ALD* alcoholic related liver disease, *ALT* alanine aminotransferase, *AST* aspartate aminotransferase, *AUROC* area under the receiver operating characteristic curve, *BMI* body mass index, *CHC* chronic hepatitis C, *CHB* chronic hepatitis B, *CLD* chronic liver disease, *FSI* fasting serum insulin, *GGT* gamma-glutamyltransferase, *MAFLD* metabolic dysfunction-associated fatty liver disease, *MASLD* metabolic dysfunction-associated steatotic liver disease, *NAFLD* non-alcoholic fatty liver disease

### Serum Biomarker Scores

#### Fatty Liver Index (FLI)

The FLI is an index score which was proposed by Bedogni et al*.* in 2006 and was developed using ultrasound as the reference modality in patients with and without suspected liver disease [36]. The FLI consists of four components, i.e. BMI, triglycerides, waist circumference, and gamma-glutamyltransferase (GGT), and has a total score range from 0 to 100, with scores < 30 ruling out fatty liver and ≥ 60 ruling it in (Table 1) [36, 37]. Regarding the diagnostic accuracy of FLI, its initially reported area under the receiver operating characteristic curve (AUROC) was 0.84 [36]. Further validation of the FLI by Fedchuck et al*.* in a cohort of patients with biopsy diagnosed NAFLD documented a similar AUROC of 0.83 for scores > 60, whereas lower sensitivity and specificity were reported at 76% and 87%, respectively [38]. Given the variation in diagnostic criteria between NAFLD and MAFLD as highlighted earlier, recent studies have further assessed the validation of FLI in patients with MAFLD [39, 40]. Indeed, the FLI was validated for predicting MAFLD in a study which assessed 278 patients by a combination of laboratory and abdominal computed tomography (CT) measures, and reported lower AUROC (0.78), sensitivity (71%) and specificity (71%) compared to NAFLD cohorts [34]. This was further corroborated by Han et al*.* amongst patients with MAFLD, where the reported AUROC for FLI was 0.79 [40]. In a recent multicenter study conducted to validate non-invasive scores on a cohort of 374 patients diagnosed according to the MASLD criteria, the FLI demonstrated a lower pooled diagnostic performance compared to previously reported cohorts of NAFLD and MAFLD, as indicated by an AUROC of 0.70, although a significantly higher sensitivity of 96% was reported [22•]. However, the generalizability of the findings from this study is limited by the recruitment of participants from secondary care centers, which may account for the suboptimal performance of the test, which was not initially designed for use in high-risk populations. Despite the diagnostic reproducibility of the FLI which makes it a simple and cost-effective tool for screening in clinical settings, its use in clinical practice is constrained by its limited ability to distinguish the severity of steatosis [38].

#### Hepatic Steatosis Index (HSI)

The HSI score is derived from a regression model/formula, using BMI, diabetes, and the alanine transaminase (ALT) to aspartate transaminase (AST) ratio (Table 1) [41]. In the initial validation study, which used ultrasound as the reference modality, the AUROC of HSI was 0.81, with cut-off scores of < 30 and > 36 for ruling out and ruling in steatosis, respectively [41]. The HSI had a high sensitivity (93%) for ruling out NAFLD, but low specificity (40%), whereas it had a high specificity (92%) for detecting NAFLD, but low sensitivity (46%) [41]. Further studies have confirmed the reproducibility of the HSI accuracy in NAFLD cohorts with AUROC of 0.79–0.82 [32, 38]. Moreover, the HSI has been also validated in CT-diagnosed MAFLD, with AUROC of 0.78 and higher sensitivity (72%) for detecting MAFLD [40]. However, a study by Koot et al*.* demonstrated that the HSI score shows decreased accuracy (AUROC of 0.68, with 67% sensitivity, and 62% specificity) amongst children with obesity [42]. Using the cut-off > 36, the diagnostic performance of HSI was also evaluated in a biopsy-proven MASLD cohort, yielding a reported AUROC of 0.80 with 96% sensitivity and 44% specificity for detecting steatosis [22•]. This performance is comparable to that observed in NAFLD and MAFLD cohorts. However, similar to the FLI, the HSI poorly distinguishes the severity of steatosis [38].

#### SteatoTest

SteatoTest is a biomarker panel published in 2005 for prediction of liver steatosis in patients with chronic liver disease, including patients with hepatitis, alcoholic liver disease (ALD) and NAFLD [43]. The model defining the SteatoTest includes 12 components/parameters, namely ALT, α2-macroglobulin (A2M), apolipoprotein A-I (ApoA1), haptoglobin, total bilirubin, GGT, cholesterol, triglycerides, glucose, age, gender, and BMI (Table 1) [43]. For all the patient groups evaluated, the SteatoTest exhibited an AUROC of 0.80 for predicting > 5% liver fat content, with a cut-off > 0.3, 90% sensitivity and 54% specificity [43]. However, there were several limitations of this study which limit the applicability of the reported results, particularly the fact that patients with NAFLD were not assessed as an independent subgroup, and that all patients were assessed by either GGT and ALT or ultrasound or a combination of either, thus introducing significant variability. The SteatoTest has been further validated in patients with NAFLD with reported accuracy between 0.73–0.82, whilst in a study by Munteanu et al*.,* the SteatoTest was validated amongst biopsy-proven NAFLD and correlated with the histological staging of steatosis [35, 44, 45]. To our knowledge the SteatoTest has not been validated in any MAFLD or MASLD cohorts.

#### NAFLD Liver Fat Score (LFS)

The NAFLD LFS is derived from a formula/model which is calculated based on the following parameters: metabolic syndrome, T2DM, fasting serum insulin, AST, and the AST/ALT ratio (Table 1) [46]. This score was initially validated in a cohort of NAFLD patients diagnosed using MRE in 2009, with a reported AUROC of 0.86 and a cut-off score of -0.64 which predicted increased liver fat content (> 5%) with sensitivity and specificity of 86% and 71%, respectively [46]. That validation study also reported that the use of genetic variables could only improve < 1% of the predictive accuracy of this score [46]. Whilst NAFLD LFS has not yet been validated in MAFLD cohorts, its accuracy was further confirmed in a cohort of 324 patients with biopsy-proven NAFLD, which showed an AUROC of 0.80, with 65% sensitivity and 87% specificity [38]. The findings of the latter study also supported the use of this score in diagnosing moderate-to-severe steatosis (> 33%). In a MASLD cohort, LFS had a reported AUROC of 0.87, similar to previously discussed NAFLD groups for the diagnosis of steatosis [22•]. However, it demonstrated a lower negative predictive value and specificity at 39% and 46%, respectively [22•]. Despite the reliability and the ease of use of this score, its practical utility in clinical settings is limited by the need to measure serum fasting insulin levels and the associated economic considerations and cost-effectiveness pertaining to routine testing.

## Diagnosis of Steatohepatitis

The development of steatosis in the context of NAFLD/MAFLD/MASLD may also trigger a local inflammatory response mediated by pro-inflammatory cytokines and immune cells [25, 27]. Indeed, the inflammatory response in NAFLD/MAFLD/MASLD involves the activation of immune cells, such as macrophages, T-lymphocytes, Kupffer cells and hepatic stellate cells, in response to hepatocellular damage and the release of pro-inflammatory cytokines [27]. Such persistent hepatic inflammation and subsequent oxidative stress creates the microenvironment of steatohepatitis, which is marked by hepatic inflammation, ballooning, and hepatic injury with or without fibrosis. Oxidative stress plays a crucial role with the generation of reactive oxygen species causing subsequent damage to cellular structures [27, 47, 48]. Mitochondrial dysfunction and endoplasmic reticulum stress contribute to this oxidative stress, further exacerbating hepatocellular injury, autophagy and apoptosis [27, 47, 48]. As aforementioned, it is evident that the pathophysiology of steatohepatitis is multifaceted, involving a combination of metabolic derangements, inflammation, oxidative stress, and fibrogenesis, thus early detection of NASH/MASH is pivotal for preventing the progression to fibrosis.

### Serum Biomarkers for the Assessment of Steatohepatitis

Several biomarkers have been evaluated for the assessment of steatohepatitis, with cytokeratin-18 (CK-18) being the most widely investigated. CK-18 is an intermediate filament protein fragment which arises from apoptosis of hepatocytes, thus allowing for correlation of its serum concentration with the degree of hepatocyte damage in order to assess disease severity relating to the histological changes of steatohepatitis [13••, 32]. In the initial validation study in 2009, CK-18 was shown to be predictive of steatohepatitis in patients with NAFLD with AUROC of 0.83, 75% sensitivity and 81% specificity [49]. Many further studies have since confirmed the diagnostic performance of CK-18, with a meta-analysis of 25 studies reporting a pooled AUROC of 0.82 with 75% sensitivity and 71% specificity [50–52]. However, challenges relating to the limited availability of CK-18 measurement and discrepancies in suggested cut-off values across studies have collectively diminished its practical utility in clinical settings [33•]. Furthermore, the limited sensitivity of CK-18 as a standalone marker has led researchers to combine it with other biological parameters. As such, Grigorescu et al*.* reported that the combination of CK-18 with interleukin-6 (IL-6) and adiponectin achieved an AUROC of 0.90 with 85% sensitivity and 86% specificity [53]. The rationale of such combinations of CK-18 with pro-inflammatory cytokines and/or adipokines are based on the underlying pathophysiologic mechanisms of steatohepatitis [54–56], since most patients are known to also have obesity and obesity related low-grade inflammation with dysregulated adipokine profiles (e.g., lower circulating levels of adiponectin which is an anti-inflammatory adipokine) [25]. For example, as a standalone biomarker, adiponectin exhibits an AUROC of 0.71 for diagnosing steatohepatitis; however, when combined with CK-18 and interleukin-8 (IL-8), this AUROC increases to 0.90 [53, 57]. Other key adipokines, such as leptin, are potential biomarkers of steatohepatitis, although further studies are needed to validate their diagnostic performance [32]. Additional pro-inflammatory cytokines have also been explored as steatohepatitis biomarkers, demonstrating moderate accuracy for discriminating between steatohepatitis and simple steatosis (e.g., CXCL10 with an AUROC of 0.68) [54]. Similarly, tumour necrosis factor-α (TNF-α) and IL-8 have shown moderate accuracy with sensitivity and specificity ranging from 65%-72% and 68–76%, respectively, whilst the combination of pyroglutamate to these two markers can increase both the sensitivity and specificity to 91% and 87%, respectively [55]. Fibroblast growth factor 21 (FGF21) which is secreted by the liver (hepatokine) is another biomarker explored for steatohepatitis. As such, Shen et al*.* reported that FGF21 had an AUROC of 0.62 for diagnosing MASH, with moderate to low positive and negative predictive values [56]. However, when combined with CK-18, these positive and negative predictive values are increased to 82% and 74%, respectively [56].

Moreover, several predictive models, incorporating both clinical and laboratory parameters, have been proposed for the diagnosis of steatohepatitis (Table 2). These include the HAIR model (hypertension, increased ALT, and insulin resistance), the oxNASH (13-hydroxyl-octadecadienoic acid/linoleic acid ratio, age, BMI, and AST), the Palekar score (age, sex, AST, BMI, AST/ALT ratio, and hyaluronic acid), the NAFIC score (ferritin, insulin, and type IV collagen 7 s), and NashTest (age, sex, height, weight, serum levels of triglycerides, cholesterol, α2-macroglobulin, apolipoprotein A1, haptoglobin, GGT, ALT, AST, and total bilirubin) (Table 2) [58–62]. Amongst these, the HAIR model exhibited a promising AUROC of 0.90 for predicting steatohepatitis, with 80% sensitivity and 89% specificity [58]. However, this performance was validated in a relatively small cohort of 26 NASH patients. The oxNASH score, which was developed in 2010, showed a correlation with histological features of inflammation and ballooning, achieving AUROC values between 0.72 and 0.74 [59, 63]. In comparison, the Palekar score, which was developed in 2006, demonstrated an AUROC of 0.76 for distinguishing steatohepatitis from simple steatosis, with moderate sensitivity (74%) and specificity (66%) [60]. For the NAFIC score, Sumida et al*.* reported an AUROC ranging from 0.78 to 0.85 for predicting NASH among 619 biopsy-proven NAFLD cases [61]. Finally, in a meta-analysis involving 494 patients with obesity and NASH, the NashTest exhibited a weighted AUROC of 0.84, with 93% sensitivity and 34% specificity [44]. These findings highlight the potential of these scores as tools for predicting and distinguishing steatohepatitis. However, it is essential to acknowledge that further validation and assessment in larger and more diverse cohorts are warranted. Indeed, many of these models, including those discussed, often rely on data from small and highly selected populations, such as patients with severe obesity, whilst external validation is lacking, especially within MAFLD and MASLD cohorts (Table 2).

**Table 2:** Diagnostic performance and limitations of serum biomarker scores for steatohepatitis assessment

**Steatohepatitis scores**	**Components**	**Sensitivity**	**Specificity**	**AUROC**	**Limitations**
**HAIR model** [58]	Hypertension, increased ALT, and insulin resistance	80%	89%	0.90	High cost; limited applicability to severely obese patients; requires further external validation
**oxNASH** [59]	13-hydroxyl-octadecadienoic acid/linoleic acid ratio, age, BMI, and AST	84%	63%	0.74	Modest accuracy; interpretation of BMI might vary across different ethnicity; influenced by age and BMI; not routinely available in clinic settings
**Palekar score** [60]	Age, sex, AST, BMI, AST/ALT ratio, and hyaluronic acid	74%	66%	0.76	Modest accuracy; influenced by age and sex; requires further external validation with larger cohorts
**NAFIC score** [61]	Ferritin, insulin, and type IV collagen 7 s	60%	87%	0.78	Modest accuracy; high cost; not routinely available in clinic settings; requires further external validation
**NashTest** [62]	Age, sex, height, weight, serum levels of triglycerides, cholesterol, α2-macroglobulin, apolipoprotein A1, haptoglobin, GGT, ALT, AST, and total bilirubin	33%	94%	0.79	Modest accuracy; high cost; influenced by age and sex; not routinely available in clinic settings; requires further external validation

*ALT* alanine aminotransferase, *AST* aspartate aminotransferase, *AUROC* area under the receiver operating characteristic curve, *BMI* body mass index, *GGT* gamma-glutamyltransferase

## Diagnosis and Staging of Liver Fibrosis

Non-invasive serum biomarkers and scores can be used to risk stratify patients with NAFLD/MAFLD/MASLD to exclude significant fibrosis and identify high risk patients who may need specialist referral. The diagnostic performance of the most commonly used fibrosis scores and biomarker panels is summarised in Table 3. Of note, the conventional belief that only steatohepatitis carries the risk of fibrosis progression is challenged by evidence demonstrating that both simple steatosis and NASH/MASH can evolve into hepatic fibrosis [23•, 64]. Overall, the progression of fibrosis in patients with NAFLD/MAFLD/MASLD and NASH/MASH is highly variable and ranges over time (e.g., ranging between 20–40% over a period of 3–6 years) [23•]. Both genetic and epigenetic determinants have been implicated in the risk of hepatic fibrosis progression. The significance of fibrosis stage as a robust predictor of overall mortality and cardiovascular risk in NAFLD/MAFLD/MASLD patients is underscored by a six-fold increase in mortality among those with cirrhosis compared to those with mild fibrosis [13••, 20]. According to the NASH Clinical Research Network, fibrosis is categorised into no or mild fibrosis (F0-1), significant fibrosis (≥ F2), advanced fibrosis (≥ F3), and cirrhosis (F4) [27, 65]. While the ultimate goal is for non-invasive fibrosis tests to provide similar information to a liver biopsy, currently none of these tests have achieved a comparable level of accuracy in assessing liver fibrosis on its own. Table 4 summarises the components, formulas and interpretation for key fibrosis scores, including aforementioned scores for steatosis and steatohepatitis.

**Table 3:** Selected key studies on the diagnostic performance of serum biomarker scores for hepatic fibrosis assessment

**Non-invasive fibrosis scores**	**Population [Ref.]**	**Cut-offs**	**Sensitivity**	**Specificity**	**PPV**	**NPV**	**AUROC**
**Fibrosis-4** (age, platelet count, AST, and ALT)	n = 541 with NAFLD [67]	≤ 1.3 ≥ 2.67	74% 33%	71% 98%	43% 80%	90% 83%	0.80
	n = 452 with NAFLD [68]	≥ 1.5	75%	67%	58%	82%	0.78
	n = 328 with NAFLD [69]	≤ 1.3 ≥ 2.67	56% 22%	56% 87%	22% 27%	85% 84%	0.54
	n = 1910 with NAFLD [71]	≥ 2.67	32%	96%	66%	85%	0.80
	n = 417 with MAFLD [73]	> 1.05	74%	62%	54%	80%	0.74
	n = 293 with MAFLD [74]	> 1.02	58%	58%	18%	90%	0.60
	n = 109 with MASLD [22•]	≤ 1.3 ≥ 2.67	64% 22%	83% 99%	52% 83%	89% 82%	0.82
	n = 6297 with MASLD [12]	≤ 1.3 ≥ 2.67	76% 26%	70% 97%	81% 93%	63% 44%	0.80
**AST-to-platelet ration index** (AST, platelet count)	n = 452 with NAFLD [68]	> 0.56	62%	76%	61%	76%	0.75
	n = 682 with NAFLD [71]	> 1.5	33%	91%	56%	79%	0.75
	n = 100 with NAFLD [81]	> 1.45	52%	89%	50%	90%	0.78
	n = 417 with MAFLD [73]	> 0.42	81%	44%	47%	80%	0.67
	n = 293 with MAFLD [74]	> 0.3	37%	84%	26%	89%	0.62
	n = 109 with MASLD [22•]	< 0.5 ≥ 1.0	53% 16%	91% 99%	61% 83%	87% 81%	0.85
**NAFLD fibrosis score** (BMI, age, hyperglycaemia, AST/ALT ratio, albumin, and platelets)	n = 733 with NAFLD [83]	≤ -1.455 ≥ 0.675	82% 51%	77% 98%	56% 90%	93% 85%	0.82
	n = 126 with NAFLD [84]	≤ -1.455 ≥ 0.675	- 96%	- 84%	- 70%	- 98%	0.92
	n = 138 with NAFLD [85]	≤ -1.455 ≥ 0.675	- 22%	- 100%	- 100%	- 81%	0.68
	n = 122 with NAFLD [86]	≥ 0.675	9%	98%	50%	83%	0.84
	n = 417 with MAFLD [73]	> -2.1	71%	67%	56%	79%	0.72
	n = 293 with MAFLD [74]	> 0.16	52%	76%	25%	91%	0.68
	n = 109 with MASLD [22•]	≤ -1.455 ≥ 0.675	86% 29%	32% 88%	25% 40%	89% 82%	0.67
**BARD Score** (BMI > 28 kg/m^2^, AST/ALT ratio > 0.8, diabetes)	n = 827 with NAFLD [89]	0–1 2–4	- -	- -	- 43%	- 96%	0.81
	n = 126 with NAFLD [84]	0–1 2–4	- 89%	- 89%	- 69%	- 97%	0.92
	n = 138 with NAFLD [85]	0–1 2–4	- 51%	- 77%	- 45%	- 81%	0.67
	n = 417 with MAFLD [73]	> 2	42%	78%	53%	69%	0.61
	n = 293 with MAFLD [74]	> 3	29%	88%	27%	89%	0.59

*ALT* alanine aminotransferase, *AST* aspartate aminotransferase, *AUROC* area under the receiver operating characteristic curve, *BMI* body mass index, *MAFLD* metabolic dysfunction-associated fatty liver disease, *MASLD* metabolic dysfunction-associated steatotic liver disease, *NAFLD* non-alcoholic fatty liver disease, *NPV* negative predictive value, *PPV* positive predictive value

**Table 4:** Serum biomarker scores for the non-invasive assessment of NAFLD/MAFLD/MASLD

**Non-invasive scores**	**Components**	**Formula**	**Interpretation**
**Fatty Liver Index** [37]	BMI, triglycerides, waist circumference, GGT	FLI = [e^0.95xloge (TG)+0.139×BMI+0.718xloge (GGT) +0.053×waist circumference−15.745^] / [1 + e^0.953×loge (TG)+0.139×BMI+0.718×loge (GGT)+0.053×waist circumference–15.745^] × 100	Rules out steatosis < 30 Rules in steatosis ≥ 60
**Hepatic Steatosis Index** [41]	BMI, diabetes, AST/ALT ratio	HSI = 8 x (ALT/AST) + BMI + (2, if diabetes mellitus) + (2, if female)	Rules out steatosis < 30 Rules in steatosis > 36
**SteatoTest** [45]	ALT, α2-macroglobulin, apolipoprotein A-I, haptoglobin, total bilirubin, GGT, cholesterol, triglycerides, glucose, age, gender, and BMI	This test is exclusively available online, as a patented algorithm. Available on: www.biopredictive.it	Range 0–1 Rules in steatosis > 0.38
**NAFLD Liver Fat Score** [46]	metabolic syndrome, type 2 diabetes, FSI, AST, and AST/ALT ratio	NAFLD LFS = -2.89 + 1.18 × metabolic syndrome (Yes = 1/No = 0) + 0.45 × Type 2 diabetes mellitus (Yes = 2/No = 0) + 0.15 × fasting serum insulin (mU/L) + 0.04 × fasting AST (U/L) – 0.94 × AST/ALT	Rules out steatosis ≤ -0.64 Rules in steatosis > -0.64 Moderate/severe steatosis ≥ 0.16
**Fibrosis-4** [78]	Age, platelet count, AST, ALT	FIB-4 Score = (Age* x AST)/ (Platelets x √ (ALT))	Rules out fibrosis ≤ 1.3 Advanced fibrosis ≥ 2.67
**AST-to-platelet ratio index** [82]	AST, platelet count	APRI = (AST in IU/L) / (AST upper limit of normal in IU/L) / (Platelets in 10^9^/L)	Rules out fibrosis < 0.3 Rules out cirrhosis < 0.5 Significant fibrosis > 1.5
**NAFLD fibrosis score** [83]	BMI, age, hyperglycaemia, AST/ALT ratio, albumin, and platelets	NAFLD Score = -1.675 + (0.037 × age [years]) + (0.094 × BMI [kg/m^2^]) + (1.13 × IFG/diabetes [yes = 1, no = 0]) + (0.99 × AST/ALT ratio)—(0.013 × platelet count [× 10^9^/L])—(0.66 × albumin [g/dl])	No to moderate fibrosis < -1.455 Advanced fibrosis > 0.675
**BARD Score** [89]	BMI > 28 kg/m^2^, AST/ALT ratio > 0.8, diabetes	BMI > 28 kg/m^2^ = 1 point AST/ALT ratio > 0.8 = 2 points Diabetes = 1 point	Low risk of advanced fibrosis 0–1 High risk of advanced fibrosis 2–4

*Caution in those with age > 65 years, score less reliable

*ALT* Alanine Aminotransferase, *AST* Aspartate Aminotransferase, *BMI* body mass index, *FSI* fasting serum insulin, *GGT* gamma-glutamyltransferase, *IFG* impaired fasting glucose

### Serum Biomarker Scores for Liver Fibrosis

#### Fibrosis-4 (FIB-4)

FIB-4 is a commonly used biomarker score, consisting of age, platelet count, AST, and ALT, which was first proposed in 2006 for the assessment of fibrosis severity in hepatitis C patients with human immunodeficiency virus (Table 4) [66]. This score has later been validated in multiple and ethnically diverse NAFLD cohorts with consistent accuracy, leading to FIB-4 being one of the two serum scores endorsed by European Association for the Study of the Liver (EASL) for the non-invasive assessment of fibrosis [67–70]. In a 2017 meta-analysis of patients with NAFLD, FIB-4 had an AUROC of 0.80 for advanced fibrosis and 0.85 for cirrhosis [71]. Different cut-offs have been suggested to improve the specificity and positive predictive value of FIB-4. As such, McPherson et al*.* reported that a FIB-4 score of > 3.25 predicted advanced fibrosis with 98% specificity and 75% positive predictive value [72]. However, lower sensitivity (26% vs. 85%) and negative predictive value (85% vs 95%) for advanced fibrosis were described for a lower cut-off of > 1.3 compared to > 3.25, respectively [72]. Recently, FIB-4 has been evaluated in patients with MAFLD for predicting advanced fibrosis, although slightly lower accuracies and different cut-offs have been demonstrated compared to NAFLD cohorts (Table 3) [73, 74]. FIB-4 has also been assessed in patients with MASLD, and, by using traditional cut-offs (> 2.67) for detecting fibrosis, good diagnostic performance was demonstrated (AUROC of 0.80–0.82 and 97–99% specificity) [12, 22•]. However, this was challenged by the findings of a study by Green et al*.* which showed that the utilisation of currently accepted cut-offs of FIB-4 is inadequate for detecting advanced fibrosis amongst patients with MASLD and severe obesity, with a reported AUROC of 0.57 [75]. As such, these authors proposed a revised cut-off of > 1.53 which increased the AUROC to 0.69, although it remained significantly lower than results discussed earlier [75]. Furthermore, Roh et al*.* have also showed that combining the FIB-4 with sonographic results improves the diagnostic accuracy of ruling in patients with advanced fibrosis [76]. Overall, FIB-4 performs best at distinguishing advanced fibrosis and cirrhosis from no/mild fibrosis, but not at discriminating between intermediate stages of fibrosis [32, 77]. Despite the feasibility and reproducibility of this score, studies have demonstrated limitations of using the FIB-4 in patients > 65 years where it has been shown to result in a high false positive rate [78, 79]. Accordingly, this has recently led to the introduction of age-based FIB-4 cut-offs (> 2 for ages > 65 years, and > 2.67 for ages > 70 years) [78, 79].

#### AST-to-platelet Ratio Index (APRI)

APRI is calculated as [(AST/upper limit of normal) / platelet count] × 100, and, similar to the FIB-4, it was initially designed as a simple calculation for diagnosing fibrosis severity in patients with chronic hepatitis C (Table 4) [80]. Studies have demonstrated moderate accuracy of APRI for predicting fibrosis in patients with NAFLD (Table 3) [68, 71, 81, 82]. In a meta-analysis by Xiao et al*.,* the APRI had an AUROC of 0.75 for discriminating advanced fibrosis and cirrhosis, although pooled sensitivities were low [71]. An APRI threshold of 1.5 had a 33% and 91% sensitivity and specificity, respectively, for advanced fibrosis [71]. This score has demonstrated consistency of its high negative predictive value, positioning it as a reliable tool in differentiating between patients with advanced fibrosis and no fibrosis, although it fails to discriminate between intermediate stages of fibrosis [32, 77]. Upon assessment in patients diagnosed with MAFLD, APRI demonstrated notably diminished accuracy, with AUROC values of 0.62 and 0.67 in two independent studies and lower cut-off values 0.3 to 0.42 compared to NAFLD cohorts [73, 74]. In a study by Wu et al*.,* the reported negative predictive value was below 80%, whilst the positive predictive value was around 50% at any cut-off value assessed [73]. However, when evaluated in patients with MASLD, a high diagnostic performance was reported with an AUROC of 0.85 (sensitivity 16%, specificity 99%) for detecting advanced fibrosis [22•]. Nevertheless, these findings are likely limited by the confounding effects of sampling a high-risk cohort which may not accurately reflect the characteristics of the general population at risk of MASLD [22•]. This underscores the need to avoid indiscriminately assuming the equal applicability of this scoring system across the three diagnostic entities (NAFLD, MAFLD and MASLD).

#### NAFLD Fibrosis Score (NFS)

The NFS calculation incorporates BMI, age, hyperglycaemia, AST/ALT ratio, albumin, and platelets, and was initially developed for 733 patients with biopsy-proven NAFLD (Table 4) [83]. Similar to the FIB-4, the NFS is also endorsed by the EASL and constitutes one of the most frequently utilised scoring systems for assessing fibrosis severity [70]. The NFS has been widely validated across multi-ethnic NAFLD cohorts, with reproducible accuracy in identifying those at both low and high risk of advanced fibrosis (Table 3) [72, 79, 84–86]. In a multi-centre study by Angulo et al*.,* NFS had an AUROC of 0.82, with a cut-off < -1.455 for low probability of advanced fibrosis exhibiting 82% sensitivity and 77% specificity, while a cut-off of > 0.675 predicted advanced fibrosis with 51% sensitivity and 98% specificity [83]. Similarly to other fibrosis scores, NFS exhibited moderate reported accuracy when validated in MAFLD cohorts, and much lower accuracy in a recently reported MASLD cohort (Table 3). The major limitation of the NFS lies in its susceptibility to be influenced by BMI and age which can lead to a high false positive rate [77], whilst it is also inaccurate in patients with an otherwise significantly affected platelet count (e.g., those with asplenia or a trans-jugular intrahepatic portosystemic shunt) [87]. In this context, a study by Dabbah et al*.* demonstrated that NFS (> -1.455) is more specific than FIB-4 for predicting advanced fibrosis in lean patients with MASLD (AUROC of 0.85 and 0.79, respectively) [88].

#### BARD Score

The BARD score was developed in a retrospective study involving 827 patients with biopsy-proven NAFLD and is an easily calculated index score which is derived by the sum of three parameters, namely BMI > 28 kg/m^2^ (1 point), AST/ALT ratio > 0.8 (2 points) and diabetes (1 point) [89]. Thus, the BARD score ranges from 0 to 4, with a score of 0–1 representing low risk of advanced fibrosis and a score of 2–4 high risk of advanced fibrosis (Table 4). The initially reported AUROC of the BARD score was 0.81, with 96% negative predictive value, but only 43% positive predictive value [89]. However, in subsequent validation studies amongst patients with NAFLD, a similar diagnostic performance was not achieved, with one study reporting an AUROC of 0.73 with 77% negative predictive value, whilst another meta-analysis demonstrated a pooled AUROC of 0.73 for advanced fibrosis in NAFLD patients (Table 3) [71, 84–86]. Moreover, the BARD score performance has been reported to be even lower when validated in patients with MAFLD with an AUROC of 0.59–0.61, 27–53% positive predictive value and 69–89% negative predictive value [73, 74]. To the best of our knowledge the BARD score has not been validated yet in any MASLD cohorts. Although the BARD score can be readily calculated using routine clinical data, its utility is constrained by its suboptimal reproducibility. Of note, the inclusion of BMI in the calculation of the BARD score may also introduce another limitation on its reliability, particularly amongst certain ethnicities that appear to be more susceptible to obesity-related comorbidities (e.g., patients of South Asian origin presenting with steatotic liver disease at lower BMI levels); thereby, potentially compromising the applicability of the BARD score across diverse demographic groups [32, 77].

**Fig.3:**
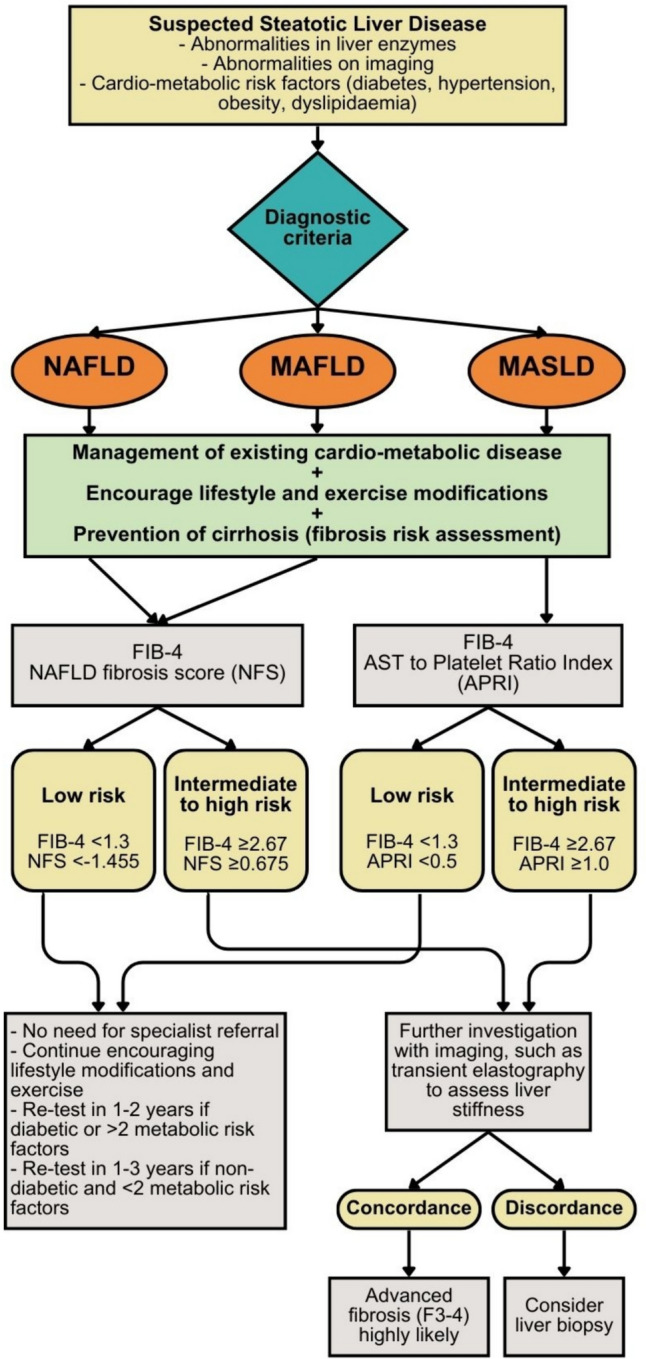
A suggested simplified algorithm for the use of non-invasive serum biomarker scores for the assessment of patients across the three diagnostic entities of steatotic liver disease (NAFLD/MAFLD/MASLD) [6, 91]

Overall, among the widely utilised fibrosis related scores, FIB-4 and NFS have undergone extensive research and demonstrate notable accuracy, particularly in achieving a high negative predictive value (> 90%) which effectively rules out advanced fibrosis with consistent results. This is substantiated by a comprehensive meta-analysis encompassing over 13,000 patients, where the FIB-4 and NFS were the most accurate for diagnosing advanced fibrosis [71]. In the context of MAFLD, the APRI and BARD scores exhibit suboptimal performance, while the FIB-4 and NFS scores are more promising in predicting advanced fibrosis [73]. However, it is crucial to emphasise that additional studies are essential to validate the diagnostic accuracy of FIB-4 and NFS amongst the newly defined MASLD and to establish new cut-off thresholds. To date, only the FIB-4 and APRI have shown satisfactory diagnostic performance within MASLD cohorts. A recent study by Eren et al*.* reported limited clinical utility of both FIB-4 and NFS in excluding advanced fibrosis, particularly among lean patients with MAFLD and those with morbid obesity [90]. This underscores the importance of ongoing research to refine and tailor these scores for diverse patient populations. Moreover, although such fibrosis scores perform well in excluding advanced fibrosis and, thus, may be suitable as initial screening tools to identify low-risk patients, approximately 20% of patients fall into an indeterminate category between the rule-in and rule-out cut-offs, necessitating further assessments [71]. As such, current guidelines recommend non-invasive imaging to enhance the predictive accuracy of these scores and reduce the need for liver biopsy [6, 70]. Figure 3 demonstrates a simplified algorithmic approach for the use of serum biomarker scores for the assessment of patients across the three diagnostic entities [6, 91].

### Serum Biomarkers and Biomarker Panels

The most severe stages of the NAFLD/MAFLD/MASLD disease spectrum includes fibrosis and cirrhosis. Indeed, preceded by steatohepatitis, which is characterised by persistent inflammation and oxidative stress, a microenvironment conducive to the activation of hepatic stellate cells is created [27]. These activated hepatic stellate cells play a pivotal role in the fibrogenic process by transforming into myofibroblasts [23•, 27]. In turn, the latter contribute to the excessive production and deposition of extracellular matrix proteins—particularly collagen which is the main component of fibrotic scars—thus, resulting in fibrous scar tissue in the hepatic parenchyma with subsequent vascular remodelling [23•, 27]. Accordingly, serological markers that are products or by-products related to collagen as a result of this pathophysiological process can be assessed to detect fibrosis [23•, 92]. The most validated of such fibrosis biomarkers include the amino-terminal propeptide of procollagen type 3 (PIIINP), hyaluronic acid, and tissue inhibitor of metalloprotinease-1 (TIMP1) [32, 93–97]. Among these biomarkers, PIIINP acts as a direct measure of type 3 collagen formation in tissues and has been demonstrated to have good diagnostic performance for diagnosing significant hepatic fibrosis (AUROC of 0.81) [93]. Hyaluronic acid performs a main role in the structural formation of extra-cellular matrix and has been shown to have AUROC of 0.89 for advanced hepatic fibrosis, with 85% and 80% sensitivity and specificity, respectively [98]. TIMP1 regulates the matrix metalloproteinases and inhibits the degradation of the extracellular matrix, thus reflecting tissue matrix remodelling. To date, TIMP1 has shown moderate performance for diagnosing hepatic fibrosis (AUROC of 0.74), while it has a much higher accuracy for discriminating steatohepatitis with an AUROC of 0.97, 97% sensitivity and 100% specificity [77, 93]. Another fibrosis-related biomarker is the N-terminal propeptide of type 3 collagen (PRO-C3), which is a fragment of collagen released during the process of fibrosis and detects the synthesis of type 3 collagen [92]. In a study by Nielsen et al*.* amongst 570 biopsy-proven NAFLD, PRO-C3 yielded a reported AUROC of 0.73 for advanced fibrosis, with significant correlation with the fibrosis stage [94]. This has been further confirmed in several studies of PRO-C3 which reported AUROC of 0.75–0.83 for advanced hepatic fibrosis and 0.76 for cirrhosis [95, 96]. Due to its correlation with the extent of hepatic fibrosis, PRO-C3 proves valuable for monitoring the efficacy of anti-fibrotic treatments [13••]. However, it must be noted that individuals with advanced hepatic fibrosis may exhibit inactive disease, leading to reduced collagen production and consequently normal levels of PRO-C3 [97]. This may potentially lead to a misleading interpretation, falsely suggesting that these patients do not harbor advanced hepatic fibrosis. Therefore, comprehensive clinical assessment and consideration of multiple indicators are essential to accurately evaluate the fibrotic status of the liver in such cases.

Overall, none of the aforementioned biomarkers exhibit adequate accuracy on its own for diagnosing hepatic fibrosis. Consequently, to enhance their diagnostic precision, composite biomarker panels have been developed, which integrate multiple fibrosis-related biomarkers with other pertinent variables [32, 77]. This approach aims to leverage the strengths of individual biomarkers and improve the overall diagnostic performance, providing a more comprehensive and reliable assessment of the underlying fibrotic status of the liver. Among these, the most commonly utilised biomarker panel is the enhanced liver fibrosis (ELF) test which combines hyaluronic acid, PIIINP and TMP1 with age [99]. The ELF test, using a threshold of 9.8, has been shown to have good diagnostic accuracy with an AUROC of 0.83, 65% sensitivity, and 86% specificity [99]. This has been further confirmed by other studies amongst patients with NAFLD, including predicting liver-related morbidity and mortality in patients with chronic liver disease [100–102]. However, the ELF test has certain limitations, relating to the influence of age on the reported results which requires further validation, as well as to its limited applicability in patients with concurrent diseases that are associated with increased collagen turnover (e.g., interstitial lung disease) since these can result in false positive results [77, 87]. To the best of our knowledge, the ELF panel has not been yet validated amongst patients where the MAFLD diagnostic criteria have also been applied. To date, only one study has reported on the application of the ELF test in patients with MASLD [12]. In that study by Younossi et al*.,* the ELF panel (> 9.8 cut-off) had a similar diagnostic performance to NAFLD cohorts, with an AUROC of 0.80 for detecting advanced fibrosis (69% sensitivity, 78% specificity, 88% positive predictive value, and 53% negative predictive value) [12].

Other similar biomarker panel models include the ADAPT score, the FibroTest, and a novel machine learning algorithm (MLA) score developed based on PRO-C3 [23•, 77]. In a study by Daniels et al*.,* the ADAPT score exhibited superior performance compared to established fibrosis scores (APRI, FIB-4, NFS, and BARD) in identifying advanced hepatic fibrosis, achieving an AUROC of 0.88 amongst a cohort of NAFLD patients [96]. Recent studies have shown that a combination of the ADAPT score with liver stiffness measurements facilitated through elastography can be used to rule out advanced hepatic fibrosis [103]. The MLA score was developed by Feng et al*.* in 2021, and incorporates BMI, PRO-C3, type-IV collagen, and AST-to-GGT ratio, demonstrating a high AUROC of 0.89 for the detection of significant hepatic fibrosis [104]. In addition, the FibroTest has demonstrated favourable diagnostic accuracy for advanced hepatic fibrosis in NAFLD patients; however, its performance across studies has not consistently yielded uniform results [105]. Collectively, despite the promise shown by these fibrosis-related biomarkers, challenges such as high cost, restricted availability, and the need for validation across large and diverse cohorts have hindered their widespread adoption in routine clinical settings [23•, 32, 77]. These considerations underscore the ongoing need for further research and validation—including validation in patients diagnosed based on the MASLD diagnostic criteria—in order to establish the broader applicability and utility of these biomarkers in the detection and risk stratification of hepatic fibrosis in routine clinical practice.

## Novel Biomarkers and Combination Scores

In recent years, several novel biomarkers and combination scores have been proposed for hepatic steatosis, steatohepatitis, and fibrosis, although their clinical availability remains limited compared to those previously discussed, with their use restricted to predominantly research settings [106••]. Recognising the dynamic nature of epigenetic markers and their pivotal role in mediating gene-environment interactions, multiple epigenetic markers have emerged as potential biomarkers for NAFLD/MAFLD/MASLD [106••, 107]. In the context of steatohepatitis, circulating microRNAs have gained attention as a biomarker for disease severity [32]. A 2018 meta-analysis evaluated miR-34a, and demonstrated a pooled AUROC of 0.78 for distinguishing between simple steatosis and steatohepatitis [108]. Similarly, other studies have investigated the diagnostic potential of miR-122, reporting pooled AUROC ranging between 0.64 and 0.70 [109, 110]. Moreover, Becker et al*.* evaluated a combination panel of circulating microRNAs (miR-122, -192, -21) alongside CK18, and reported an AUROC of 0.83 for diagnosing steatohepatitis [111]. Regarding the assessment of advanced fibrosis, plasma DNA methylation of the peroxisome proliferator-activated receptor-gamma (PPARγ) has exhibited promising diagnostic performance, with an AUROC of 0.91, with a positive and negative predictive value of 91% and 87%, respectively [112]. Another avenue of biomarkers predicting advanced fibrosis involves macrophage markers. The macrophage activation marker (sCD163) demonstrated an AUROC of 0.83 when combined with the NFS score, while the macrophage-derived deaminase marker predicted advanced fibrosis with an AUROC of 0.82 [113, 114]. Despite their initial promising performance these novel biomarkers are often derived from small cross-sectional cohorts and further studies with larger validation groups are imperative to confirm and establish their diagnostic utility.

As a polygenic disease, NAFLD/MAFLD/MASLD shares a number of genetic variants with other metabolic conditions, implicating them in disease susceptibility and progression [23•]. These genetic variants govern key pathways central to the underlying NAFLD/MAFLD/MASLD pathophysiology, such as lipid metabolism, insulin and adipokine signaling, and inflammatory regulation [23•, 106••]. Among the most extensively studied genetic variants associated with fibrosis progression are PNPLA3, TM6SF2 and MBOAT7 [115–117]. In a cohort study by Krawczyck et al*.* which assessed 515 patients with NAFLD for the aforementioned genotypes, these three variants demonstrated significant associations with hepatic injury [118]. Indeed, PNPLA3 was associated with a higher risk for both steatosis and hepatic fibrosis, while TM6SF2 was primarily linked with steatosis and MBOAT7 with hepatic fibrosis [118]. Furthermore, recent efforts have focused on constructing polygenic risk scores incorporating clinical parameters to enhance their predictive performance [106••]. Nevertheless, challenges persist regarding the interpretation of these genetic scores, their application for risk stratification versus risk prediction, and their limited generalisability given that the majority of genome studies are conducted on European populations [106••].

Given the intimate association of NAFLD/MAFLD/MASLD with metabolic dysfunction, there has also been a growing interest to harness metabolomics and lipidomics to distinguish between steatosis and steatohepatitis and to predict hepatic fibrosis. In this context, Caussy et al*.* demonstrated that a predictive score combining a serum metabolite panel accurately predicted advanced fibrosis in patients with NAFLD (biopsy-proven or assessed using MRI elastography) with an AUROC up to 0.94, outperforming both FIB-4 and NFS [119]. In another study, a triglyceride panel successfully differentiated between healthy individuals and those with NAFLD, as well as between steatohepatitis and simple steatosis [120]. The NASH ClinLipMet Score represents another metabolic-based combination score, incorporating five metabolites (i.e. glutamate, isoleucine, glycine, lysophosphatidylcholine 16:0, phosphoethanolamine 40:6) with PNPLA3 genotype and clinical variables, including AST and fasting insulin [121]. One of the validation studies reported that this score had an AUROC of 0.87 for distinguishing steatohepatitis from steatosis, with a moderate sensitivity of 75% [121]. Promising proteomics biomarkers for NAFLD/MAFLD/MASLD are angiopoietin-like proteins (ANGPTLs) [122]. These belong to the glycoprotein family which consists of eight members (ANGPTL1–8), which all share a common structure with specific features that makes them different in tissue expression and regulation [122]. ANGPTLs play a significant role in lipid metabolism, insulin resistance, and hormone regulation and may be an important link to the metabolic syndrome [122]. Emerging studies have demonstrated certain correlations between circulating ANGPTLs with NAFLD, although results have been inconsistent [123–125]. In a recent meta-analysis of 13 studies, pooled evidence showed that some ANGPTLs may be closely related to NAFLD, with ANGPTL8 being found at significantly higher levels in patients with NAFLD compared to healthy individuals [126]. The positive association of ANGPTL8 with the occurrence of NAFLD has also been demonstrated with respects to progression across the disease spectrum, since patients with moderate to severe NAFLD appear to have higher ANGPTL8 levels compared to patients with mild NAFLD, posing this ANGPTL as a potential marker for disease monitoring in different stages [123, 126].

Furthermore, the gut microbiome has emerged as a promising source of metabolite biomarkers, prompting clinical exploration into the utility of faecal/gut microbiota markers of disease as non-invasive diagnostic biomarkers [106••]. Loomba et al*.* and Oh et al*.* investigated this possibility, and found that incorporating faecal microbial metagenomic signatures with other clinical variables resulted in an AUROC of 0.94 and 0.91, respectively, for predicting hepatic fibrosis [127, 128]. Data suggest that a gut microbiome derived signature which may be associated with disease progression from mild/moderate NAFLD to advanced fibrosis involves an increase in Proteobacteria and Escherichia coli, with a decrease in Firmicutes and some members of Bacteroidetes such as Bacteroides vulgatus [127, 128]. However, the use of faecal samples as a biomarker source for NAFLD/MAFLD/MASLD is challenged by the confounding effects of age, sex, diet, medication, hormonal and lifestyle factors on the gut microbiota. Additionally, the complex technical methodologies for analysing the gut microbiome may limit the reproducibility of the relevant findings, whilst the associated high cost also impedes the widespread implementation in clinical practice [106••].

Hepatocellular carcinoma (HCC) is a severe complication which may arise from progressive steatohepatitis or ongoing fibrosis and cirrhosis, with an estimated 5-year survival rate of 70–75% in those with early HCC, and 15% in patients with advanced HCC [129–131]. Current guidelines recommend monitoring of high risk groups (e.g. primarily those with cirrhosis) with regular ultrasound scanning and alpha-fetoprotein (AFP) levels, which is the only biomarker approved for surveillance [70]. However, the sensitivity of ultrasound scanning is moderate, with poorer diagnostic performance in early HCC stages [132]. Many serum biomarkers have been investigated for more accurate diagnosis of NAFLD/MAFLD/MASLD-associated HCC without the need for imaging which have been discussed in detail in other reviews [133, 134]. The most common serum biomarkers for HCC diagnosis include AFP, AFP isoform L3 (AFP-L3) and des-carboxyprothrombin (DCP) [133]. Both AFP-L3 and DCP have demonstrated comparable diagnostic accuracy to AFP, with the combined use of AFP with either of the other two biomarkers improving the overall diagnostic performance when compared to each one alone [133, 135, 136]. The most reliable scoring system for HCC is the GALAD score which incorporates age, sex, AFP, lectin-bound AFP and DCP [134, 135]. In a study by Best et al*.* the GALAD score had an excellent diagnostic performance with an AUROC of 0.96, outperforming all three biomarkers (AFP, DCP, AFP-L3) when evaluated alone [135]. Whilst the GALAD score is the most promising to date, its application is limited to those with advanced HCC, with uncertain value in monitoring early HCC [134, 137]. Novel potential biomarkers are currently being investigated, including genetic and epigenetic biomarkers [133, 134]. Indeed, genomic research has identified telomerase reverse transcriptase (TERT) promoter mutations as the most common form of HCC alteration [133]. In a 2021 study, the diagnostic value of TERT mutations in the diagnosis of NAFLD-associated HCC was established, which can be used to detect early HCC even when AFP levels are normal [138]. These results may indicate the potential advantage of TERT mutations in early detection which may improve the prognosis of these patients. Macrophage apoptosis inhibitor, which is produced by tissue macrophages, has also been identified as a possible biomarker for early NASH-associated HCC detection in a number of studies [139, 140]. Additionally, Kozumi et al*.* demonstrated that serum thrombospondin 2 expression levels were significantly associated with advanced fibrosis in NAFLD patients, with HCC observed only in patients with high serum thrombospondin-2, suggesting that this may represent another potential biomarker for surveillance [141]. Despite the promise of these biomarkers, further research is needed before their clinical implementation is considered, including whether the evidence of risk factors and diagnostic markers can be applied to the new disease nomenclatures [133]. In a recent cohort study, patients meeting both NAFLD and MAFLD diagnostic criteria were noted to have similar HCC risk compared to those meeting the NAFLD criteria alone, nonetheless, more robust evidence is warranted in this area [142].

**Fig.4:**
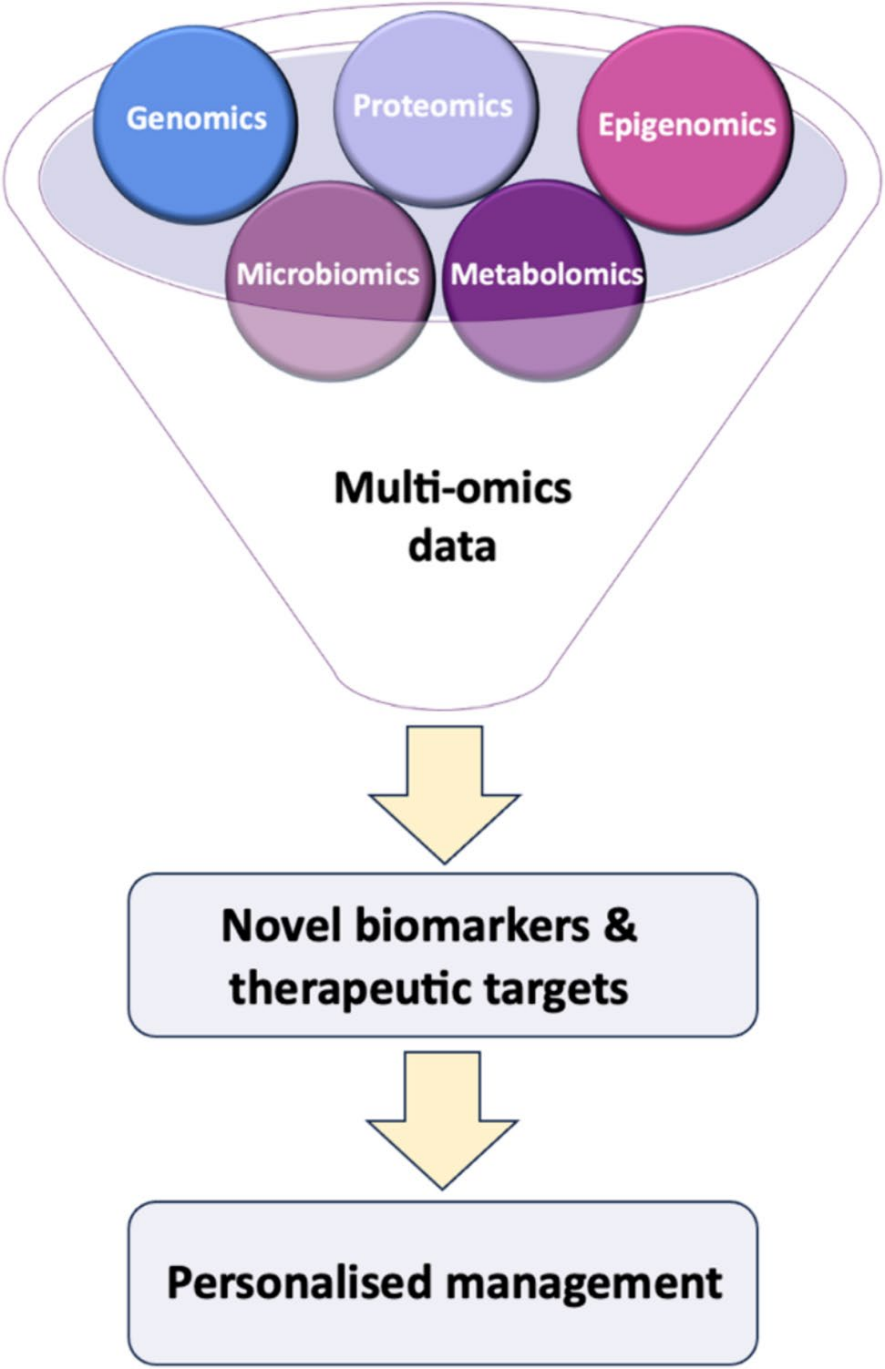
Framework for the multi-omics integration for personalised management of NAFLD/MAFLD/MASLD [106••]

Overall, the heterogeneity of NAFLD/MAFLD/MASLD underscores the exciting potential of integrating multi-omics approaches as the future of non-invasive biomarkers, offering the prospect of personalised insights (Fig. 4). However, to establish robust metabolomic signatures, reported biomarkers must undergo validation in larger and more diverse cohorts. This validation process is essential for enhancing our capacity to effectively transition from biomarker discovery to addressing the challenges of accuracy, applicability, cost, and feasibility in clinical practice.

## Conclusion

The rapidly escalating prevalence of NAFLD/MAFLD/MASLD is concerning, highlighting the urgency for early diagnosis to facilitate prompt risk stratification and management in order to prevent disease progression and complications. The past several years have witnessed a new era for the development of non-invasive scores and biomarkers for NAFLD/MAFLD/MASLD, including the potential integration of multi-omic biomarkers. However, none of these has yet been able to replace the liver biopsy as the diagnostic gold standard. Our review has emphasised the variability of these non-invasive scores and biomarkers across the diagnostic entities of NAFLD, MAFLD and MASLD, further highlighting the need to refrain from indiscriminately assuming the equal applicability of existing scientific evidence across these definitions/entities. Future studies must explore the landscape of MASLD under the newly defined disease subtypes, with consideration given to patient stratification when non-invasive scores and biomarkers are developed in clinical trials. Furthermore, previously developed scores should undergo validation using the newly defined criteria, while acknowledging the known heterogeneity of the disease by incorporating a broader range of clinical and patient profiles to ensure real-world representation. Currently, depending on availability, the recommended approach in routine clinical practice involves combining multiple tests and scores, including non-invasive imaging. However, further research is warranted to compare the efficacy of different test combinations in improving their diagnostic performance. Additionally, investigations into the cost-effectiveness of novel biomarkers or multi-omic approaches for diagnosing and monitoring disease progression are also needed.

## Data Availability

Not applicable.
